# Role of serotonin in body weight, insulin secretion and glycaemic control

**DOI:** 10.1111/jne.12960

**Published:** 2021-04-28

**Authors:** Teodora Georgescu, David Lyons, Lora K. Heisler

**Affiliations:** ^1^ Department of Anatomy Centre for Neuroendocrinology School of Biomedical Sciences University of Otago Dunedin New Zealand; ^2^ School of Physiology Pharmacology & Neuroscience University of Bristol Bristol UK; ^3^ Rowett Institute University of Aberdeen Aberdeen UK

**Keywords:** 5‐HT, body weight, glycaemic control, insulin, obesity, serotonin

## Abstract

Obesity and type 2 diabetes are key healthcare challenges of the 21st century. Subsequent to its discovery in 1948, serotonin (5‐hydroxytryptamine; 5‐HT) has emerged as a principal modulator of energy homeostasis and body weight, prompting it to be a target of weight loss medications (eg, fenfluramine, d‐fenfluramine, fenfluramine‐phentermine and sibutramine). The potential risk of off‐target effects led to these medications being withdrawn from clinical use and spurred drug discovery into 5‐HT receptor selective ligands. The serotonin 2C receptor (5‐HT_2C_R) is the primary receptor through which 5‐HT impacts feeding and body weight and 5‐HT_2C_R agonist lorcaserin was released for obesity treatment in 2012. Obese patients with type 2 diabetes prescribed medications that produce weight loss commonly observe improvements in type 2 diabetes. However, recent research has provided compelling evidence that 5‐HT_2C_R agonists produce effects on blood glucose and insulin sensitivity independent of weight loss. As such, neuroactive 5‐HT_2C_R agonists are a potential new category of type 2 diabetes medications. 5‐HT is also expressed within pancreatic β cells, is co‐released with insulin and may have a role in modulating insulin secretion. This review highlights the latest advances in the function of 5‐HT in body weight, insulin release and glycaemic control.

## 1 INTRODUCTION

The serious health consequences of diabetes are illustrated by the statistic that diabetes causes 1 death every 6 seconds and constitutes approximately 15% of human mortality worldwide.^1^ Obesity is the strongest risk factor for the development of type 2 diabetes, accounting for approximately 80% of the risk.^2^ Obesity is defined as a body mass index (BMI) greater than 30 kg m^2^ and arises when energy intake exceeds energy expenditure. The number of obese and overweight adults has almost tripled worldwide since 1975, with more than 2.1 billion adults now meeting criteria.^3^ A more striking increase in average body weight is evident in children. From 1975 to 2016, the proportion of obese children and adolescents increased from less than 1% to 5.6% in boys and 7.8% in girls.^3^

A typical strategy to treat obesity and type 2 diabetes is lifestyle modification, including reducing caloric intake and increasing physical exercise. However, this approach is commonly associated with modest and often transient weight loss, rarely exceeding 10% of body weight.^4^ Pharmacotherapies have been developed to use in conjunction with lifestyle modification to facilitate successful weight loss. In more severe cases of obesity, bariatric surgery may be recommended. Recent advances in the molecular mechanisms regulating satiety and energy homeostasis have provided new targets for drug discovery for obesity treatment. The brain has emerged as the master orchestrator of food intake and energy balance, integrating functionally relevant signals within pathways and regulatory nodes to exert coordinated control of whole body metabolism. Accumulating evidence over the past decade has also revealed a role for the brain in glycaemic control.

The focus of this review is serotonin (5‐hydroxytryptamine; 5‐HT), which is a key modulator of food intake and energy metabolism and a target of weight loss medications (eg, fenfluramine, d‐fenfluramine, fenfluramine‐phentermine, sibutramine and lorcaserin). Obese patients with type 2 diabetes prescribed medications that produce weight loss commonly observe improvements in type 2 diabetes. Recent evidence indicates that 5‐HT weight loss medications also directly improve type 2 diabetes without weight loss by reducing hepatic glucose production and increasing insulin sensitivity. 5‐HT is also expressed within the pancreatic β cells, being co‐released with insulin, and may have a role in modulating insulin secretion. These topics are discussed below.

## 2 5‐HT SYNTHESIS

The synthesis of the monoamine transmitter 5‐HT is carried out in a two‐step process that requires the essential dietary amino acid tryptophan. The first step involves the conversion of tryptophan to 5‐hydroxytryptophan. Tryptophan hydroxylase catalyses this reaction and is the rate‐limiting enzyme for the pathway.^5^ There are two isoforms of this enzyme: tryptophan hydroxylase 1 (Tph1) expressed in peripheral tissues and Tph2 expressed in the brain.^6^ In the second and final step, aromatic l‐amino acid decarboxylase converts 5‐hydroxytryptophan into 5‐HT.

5‐HT has a broad range of physiological and behavioural functions and is present in most species. In mammals, 5‐HT is synthesised in both the periphery and the central nervous system (CNS). In the periphery, 5‐HT is primarily produced by enterochromaffin cells of the gastrointestinal tract where it regulates intestinal motility.^7^ 5‐HT has also been reported in other organs and tissues and has been linked to the function of brain, liver, bone, mammary glands and pancreatic β cells.^8^, ^9^, ^10^

## 3 5‐HT RELEASE AND METABOLISM

**FIGURE 1:**
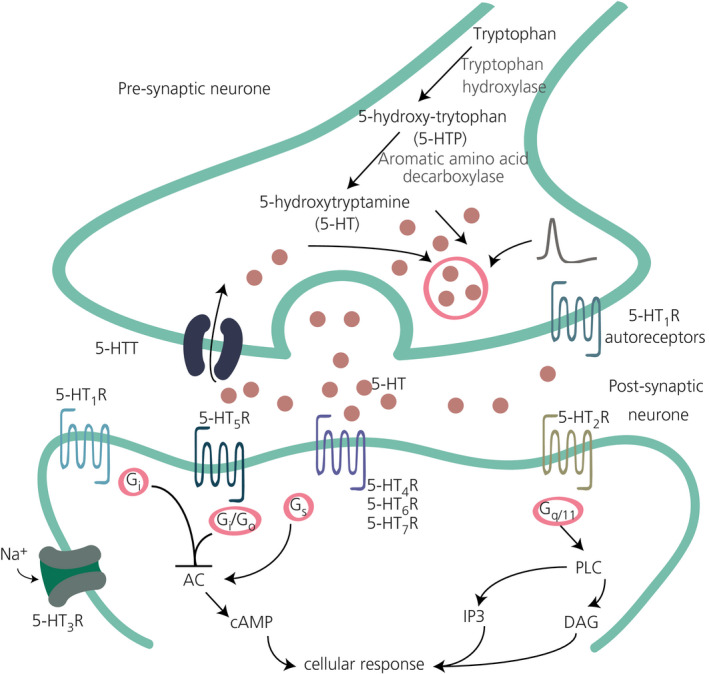
Serotonin (5‐hydroxytryptamine; 5‐HT) synthesis and neuronal signalling. 5‐HT is synthesised in the presynaptic neurone from the amino acid tryptophan. Following activation of this neurone, 5‐HT is packed into vesicles and released into the synaptic cleft, where it signals through its receptors (5‐HT_1R_ to 5‐HT_7R_). The signalling is terminated by re‐uptake of 5‐HT from the cleft by 5‐HT transporter (5‐HTT). AC, adenylate cyclase; DAG, 1,2‐diacylglycerol; IP3, inositol 1,4,5‐trisphosphate; PLC, phospholipase C

Once synthesised, 5‐HT is stored in vesicles and released through regulated exocytosis allowing it to bind to 5‐HT receptors (Figure 1). The 5‐HT transporter (5‐HTT or SERT) is responsible for the reuptake of 5‐HT. Metabolism of 5‐HT is carried out in two steps, with the first being oxidative deamination by monoamine oxidase A, yielding 5‐hydroxyindole‐3‐acetaldehyde. In the second step, aldehyde dehydrogenase catalyses the oxidation of 5‐hydroxyindole‐3‐acetaldehyde to 5‐hydroxyindoleacetic acid.

## 4 5‐HT RECEPTORS

**FIGURE 2:**
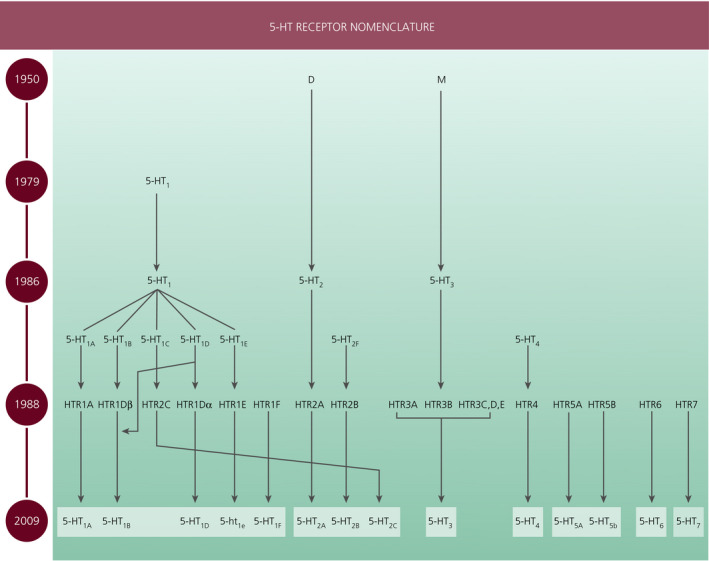
Serotonin (5‐hydroxytryptamine; 5‐HT) receptor discovery and nomenclature. 5‐HT receptors were first discovered in the 1950s and designated D and M. Subsequently, additional receptors have been identified and the receptors have been grouped into seven families based on sequence homology and effector pathways. Currently, 14 5‐HT receptors have been identified in mammals. Adapted from Sharp & Barnes^11^ and Marin et al^103^

Beginning in the 1950s, numerous 5‐HT receptors have been discovered in mammals.^11^ Following their discovery, some receptors have been renamed. 5‐HT receptors are now grouped into seven families (5‐HT_1_R to 5‐HT_7_R) based on sequence homology and intracellular effectors (Figure 2). The change in receptor nomenclature highlights the importance of the awareness of earlier names of a single receptor when reading and interpreting earlier reports. 5‐HT_3_Rs are ligand gated cation channels. All the other 5‐HT receptors comprise a G‐protein coupled with an extracellular N‐terminus, seven transmembrane domains connected by three extracellular and three intracellular loops, and an intracellular C‐terminus.^11^, ^12^ All 5‐HT receptors are expressed postsynaptically. All 5‐HT receptors are expressed in both the brain and periphery to varying degrees, with the following exceptions: 5‐HT_2C_Rs and 5‐HT_6_Rs are predominantly, if not exclusively, expressed within the CNS, whereas 5‐HT_2B_Rs are primarily expressed in the periphery.^13^

Receptors within the 5‐HT_1_ family are G_i/o_‐coupled and may also be positioned as autoreceptors and heteroreceptors. 5‐HT_5_Rs are also G_i/_o‐coupled and function to hyperpolarise cells. The 5‐HT_4_R, 5‐HT_6_R and 5‐HT_7_R families are G_s_‐coupled and their activation results in cell depolarisation. 5‐HT_2_Rs couple to G_q/11_. In addition to these canonical signalling pathways, recent evidence suggests that metabotropic 5‐HT receptors also signal through a variety of non‐canonical pathways.^11^ This is worth considering when predicting a cellular response following 5‐HT receptor activation. 5‐HT receptors are similarly distributed in vertebrates, with the following exceptions: rodents do not have 5‐ht_1e_Rs, humans do not express full‐length 5‐ht_5b_Rs, and humans and rodents exhibit a different pattern of 5‐HT_3_R and 5‐HT_6_R distribution within the forebrain.^11^ 5‐ht_1e_R and 5‐ht_5b_R are denoted with lower case appellation because a functional response in native cells has not yet been demonstrated.^13^ Isoforms of 5‐HT_2C_Rs, 5‐HT_3_Rs, 5‐HT_4_Rs and 5‐HT_7_Rs have been identified, although the specific function of these isoforms remains to be clearly established. 5‐HT_2C_Rs are the only 5‐HTR that undergo RNA editing.

## 5 β CELL 5‐HT

### 5.1 5‐HT and receptor expression

**FIGURE 3:**
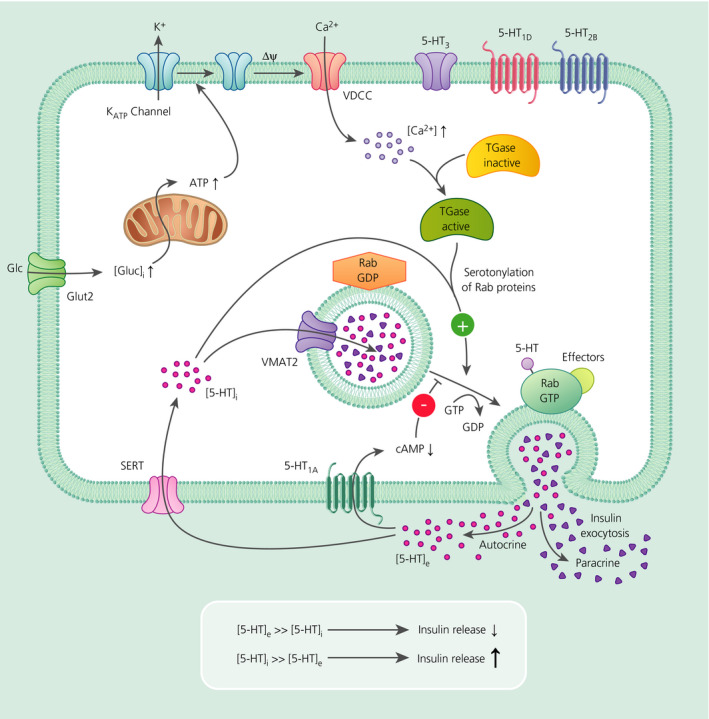
Schematic of model of exocytosis of β granules from β cells in response to serotonin (5‐hydroxytryptamine; 5‐HT). Glucose (Glu) is transported into the cell, oxidised by the mitochondria to produce ATP, which then causes the closure of the K_ATP_ channel and leads to depolarisation of the membrane. This opens the voltage‐dependent Ca^2+^ channel and the influx of Ca^2+^ ions activate transglutaminase (TGases) and serotonylate a number of proteins. Amongst these, Rab3a and Rab27a are essential for insulin release. Thereby their activation causes co‐secretion of 5‐HT and insulin. By signalling at the 5‐HT_1A_R, high extracellular 5‐HT ([5‐HT]_e_) reduces further secretion of insulin. However, this effect is dampened as 5‐HT reuptake occurs via the 5‐HT transporter (5‐HTT). When intracellular 5‐HT ([5‐HT]_i_) is substantially higher than extracellular 5‐HT ([5‐HT]_e_), this triggers another event of insulin secretion by serotonylation. This dynamic regulation of 5‐HT levels may explain the established oscillation of insulin exocytosis from glucose stimulated β cells. Adapted from Paulmann et al.^21^GLUT2, glucose transporter 2; 5‐HTT, serotonin transporter; VDCC, voltage‐dependent Ca^2+^ channel; VMAT, vesicular monoamine transporter

Evidence of 5‐HT in the pancreatic islets of Langerhans dates to studies carried out more than 50 years ago.^14^ β cells co‐release 5‐HT alongside insulin and ATP.^15^, ^16^ Expression of tryptophan hydroxylase (TPH1) in β cells indicates the capacity for de novo synthesis of 5‐HT in the islets of Langerhans.^17^, ^18^, ^19^ Mice lacking TPH1 only in β cells are glucose intolerant and secrete less insulin when fed a high‐fat diet for 6 weeks.^20^ This link is specific to β cells because TPH1 knockout in gut enterochromaffin cells does not affect insulin secretion.^21^, ^22^ Human and rodent β cells also express 5‐HTT, the transporter responsible for 5‐HT reuptake.^17^, ^19^ Research suggests that 5‐HT acts as an autocrine signal modulating β cell function and proliferation^20^, ^23^, ^24^, ^25^ (Figure 3).

The presence of 5‐HT_1A_Rs, 5‐HT_1D_Rs, 5‐HT_1F_Rs, 5‐HT_2B_Rs, 5‐HT_3_Rs and 5‐HT_5A_Rs within human islets has been revealed via a revealed via gene and/or protein expression techniques.^19^, ^25^, ^26^ Different receptors appear to be primarily expressed within specific cell types, with 5‐HT_1A_Rs, 5‐HT_1D_Rs, 5‐HT_2B_Rs and 5‐HT_3_Rs in β cells, 5‐HT_1F_Rs in α cells, and 5‐HT_5A_Rs in δ cells.^19^, ^25^, ^26^

### 5.2 Insulin secretion and β cell proliferation

5‐HT_2B_R agonists promote insulin secretion, an effect partly regulated by a rise in intracellular [Ca^2+^] and enhanced mitochondrial activity.^25^ Expression of 5‐HT_2B_Rs in mouse islets increases during pregnancy and it serves to enhance β cell proliferation during this period of high insulin demand.^23^ Pharmacological inhibition of the 5‐HT_2B_R impaired glucose tolerance and reduced the pregnancy‐associated increase in β cell mass, highlighting this receptor as a favourable target for the treatment of gestational diabetes.^23^ Regulation of insulin secretion and β cell function has also been described during lactation, and this phenomenon is dependent on 5‐HT signalling at the 5‐HT_2B_R.^27^ However, mice with a β cell‐specific knockout of 5‐HT_2B_Rs are not glucose intolerant after a 6‐week period of high‐fat diet, whereas 5‐HT_3_R knockout mice display diminishes glucose‐stimulated insulin secretion.^20^ Glucose‐dependent 5‐HT release from human β cells also acts on α cells to inhibit glucagon secretion via action at the 5‐HT_1F_R.^19^ Administration of a 5‐HT_1F_R agonist lowered glucagon secretion and improved glycaemic levels in diabetic mice.^19^ These findings suggest that pancreatic 5‐HT plays a significant role in insulin secretion via action at different 5‐HT receptors expressed on distinct cell types.^20^, ^23^

In addition to receptor‐mediated transductional processes, receptor‐independent 5‐HT signalling may also influence insulin secretion. For example, non‐canonical transductional mechanisms promote insulin exocytosis from β cells via serotonylation of Rab proteins.^21^ As demonstrated in other tissues, serotonylation relies on transamination by transglutaminases of 5‐HT to small GTPases resulting in these being constitutively active.^28^ Further supporting this receptor‐independent effect of 5‐HT, transglutaminase 2 null mice are glucose‐intolerant and show decreased levels of insulin following glucose stimulation.^29^ Walther et al^28^ propose a dynamic model in which progressive insulin and 5‐HT co‐secretion promotes high extracellular 5‐HT concentrations that, via an autocrine feedback loop, attenuates additional insulin (and 5‐HT) release through inhibitory 5‐HT_1A_Rs located on β cells. Extracellular 5‐HT is subsequently cleared via 5‐HTT. They propose that as extracellular 5‐HT levels fall and intracellular 5‐HT levels build, insulin (and 5‐HT) secretion is once more enhanced via serotonylation (Figure 3).

Although 5‐HT was identified in the islet more than half a century ago, the physiological function of 5‐HT co‐release with insulin is still largely unknown. Seemingly contradictory reports may be reconciled by differing functions of distinct 5‐HT receptors in different cell types and receptor‐independent 5‐HT action. Additional research is required to clarify the function of 5‐HT in the islet and in the modulation of insulin secretion.

## 6 BRAIN 5‐HT

### 6.1 5‐HT and receptor expression

Although comparatively little is known about the function of 5‐HT in pancreatic insulin secretion and β cell proliferation, more intense research efforts have focussed on the role of 5‐HT in the brain. CNS 5‐HT influences a wide variety of processes, such as energy balance, anxiety, mood, migraine, sleep, locomotion, circadian rhythms and aggression, amongst others. This review is focussed on the role of CNS 5‐HT in the modulation of feeding behaviour, body weight and glycaemic control.

**FIGURE 4:**
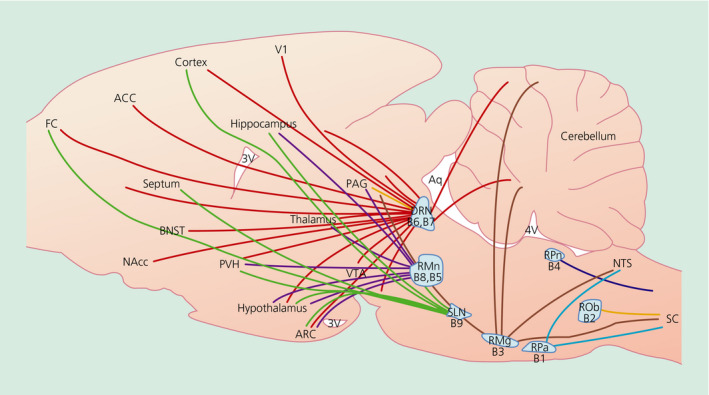
Schematic of raphe nuclei localisation and selected projections. 5‐HT neurones within the raphe nuclei cluster along the midline and broadly innervate the CNS. Displayed is a schematic of a sagittal midline brain view illustrating the relative localisation of the raphe nuclei and some selected projections. 3V, third ventricle; 4V, fourth ventricle; ACC, anterior cingulate cortex; Aq, aqueduct; ARC, arcuate nucleus of the hypothalamus; BNST, bed nucleus of the stria terminalis; RPa, raphe pallidus nucleus, B1; ROb, raphe obscurus nucleus, B2; RMg, raphe 2magnus nucleus, B3; RPn, raphe pontis nucleus, B4; RMn, median raphe nucleus, B5, B8; DRN, dorsal raphe nucleus, B6, B7; SLN, supralemniscal nucleus, B9; FC, frontal cortex; NAcc, nucleus accumbens; NTS, nucleus of the solitary tract; PAG, periaqueductal grey; PVH, paraventricular hypothalamic nucleus; SC, spinal cord; V1, primary visual cortex; and VTA, ventral tegmental area (adapted from Lam & Heisler 2007)^36^

5‐HT synthesis requires the essential amino acid tryptophan obtained from food. Tryptophan is carried by an active transport mechanism into the brain where it is converted into 5‐HT. Because tryptophan competes with the other large neutral amino acids (LNAA) for transport into the brain, the ratio of tryptophan to other LNAA is an important determinant for 5‐HT synthesis.^30^ Peripherally derived 5‐HT does not cross the blood‐brain barrier. However, recent studies reveal the presence of 5‐HTT in the brain endothelium and have linked this to transport of augmented 5‐HT from the brain into the blood, but not vice‐versa.^31^, ^32^, ^33^ Brain 5‐HT is produced in a collection of midline brainstem nuclei called raphe, meaning a ‘seam connecting two halves’. The raphe nuclei are comprised of the raphe pallidus (RPa; B1), raphe obscurus (ROb; B2), raphe magnus (RMg; B3), raphe pontis (RPn; B4), median raphe (RMn; B5, B8), dorsal raphe (DRN; B6, B7) and the supralemniscal nucleus (SLN; B9). Unlike the other raphe nuclei, the SLN is not expressed on the midline. These nuclei were previously designated B1‐B9 and project to distinct parts of the brain such that almost all brain regions are innervated by 5‐HT terminals to some extent. The cluster of caudal raphe nuclei (B1‐B4) are responsible for descending 5‐HT projections that innervate regions including the cerebellum, midbrain, pons, medulla and spinal cord, whereas the more rostral raphe nuclei (B5‐B9) give rise to ascending projections that target areas such as the cortex, hippocampus, thalamus, hypothalamus, striatum and amygdala^34^, ^35^, ^36^, ^37^ (Figure 4). The DRN is the largest raphe nuclei and distinct DRN subdivisions are relevant to energy homeostasis.^34^ The arcuate nucleus of the hypothalamus (ARC) melanocortin pro‐opiomelanocortin (POMC) and agouti‐related peptide (AgRP) neuronal populations are primary regulators of energy homeostasis and are innervated by 5‐HT fibres.^36^, ^38^, ^39^, ^40^, ^41^ The melanocortin system is required for 5‐HT weight loss medications to produce their therapeutic effect.^42^, ^43^, ^44^, ^45^, ^46^, ^47^

### 6.2 Food intake and body weight

Early research with 5‐HT revealed a strong inverse relationship between 5‐HT and food intake. Although brain 5‐HT accounts for a relatively small percentage of 5‐HT (approximately 3%), research in the 1970s revealed that brain 5‐HT is necessary for normal appetite and body weight regulation. Specifically, in back‐to‐back publications in *Science* in 1976, both Breisch et al^48^, ^49^ and Saller and Stricker^48^, ^49^ reported that selectively decreasing brain 5‐HT synthesis promoted weight gain and the development of obesity. Early pharmacological strategies to elevate brain 5‐HT to treat obesity selectively blocked 5‐HT reuptake (selective serotonin reuptake inhibitor; SSRI) and stimulated 5‐HT release.

## 7 CLINICAL AND PRECLINICAL 5‐HT MEDICATIONS

### 7.1 Medications augmenting 5‐HT bioavailability

#### 7.1.1 Fenfluramine, d‐fenfluramine and fenfluramine‐phentermine (fen‐phen)

Medications specifically targeting 5‐HT for obesity treatment were fenfluramine (Pondimin) and the d‐enantiomer of fenfluramine (d‐fenfluramine, dexfenfluramine, Redux), which stimulate the release of 5‐HT and block its reuptake. Fenfluramine and d‐fenfluramine alone and in combination with phentermine (fen‐phen) were used to treat human obesity until the late 1990s when they were withdrawn from clinical use as a result of valvular side effects in a small group of obese patients.^50^ Earlier this year, the US Food and Drug Administration (FDA) and European Medicines Agency granted or recommended granting the marketing of fenfluramine (Fintepla) for the treatment of seizures associated the severe form of epilepsy Dravet syndrome.

The primary therapeutic mechanism of fenfluramine and d‐fenfluramine in treating obesity is achieved via decreasing food intake.^51^ However, some evidence suggests that d‐fenfluramine may also increase resting and postprandial energy expenditure in obese patients.^52^ Fenfluramine was also found to have effects on glucose tolerance. For example, following a 7‐day treatment with fenfluramine, blood glucose of obese patients with type 2 diabetes was significantly lowered.^53^ The glycaemic effect may be at least in part independent of the anorexigenic effects of fenfluramine.^53^, ^54^, ^55^

Following the withdrawal of fenfluramine, d‐fenfluramine and fen‐phen for weight loss, research effort turned to establishing the receptors underpinning both the off‐target and therapeutic effects. The receptor implicated in the adverse effect is peripheral 5‐HT_2B_Rs. Compelling evidence indicating that brain 5‐HT_2C_Rs are required for the therapeutic effect was provided by transgenic mouse studies. Specifically, 5‐HT_2C_R knockout mice do not respond to the anorectic effect of d‐fenfluramine.^42^, ^56^ Given that the 5‐HT_2C_Rs are expressed in a variety of brain regions where they produce an array of behavioural effects, additional studies aimed to define the specific subset of 5‐HT_2C_Rs and the circuit through which d‐fenfluramine reduces feeding. 5‐HT_2C_Rs are co‐expressed with ARC POMC neurones and d‐fenfluramine and 5‐HT_2C_R agonists increase POMC neurone activity.^47^ In mice null for 5‐HT_2C_Rs, re‐expression of 5‐HT_2C_Rs only in POMC neurones restored the anorectic effect of d‐fenfluramine.^42^ Illustrating the necessity of POMC peptides the effect of d‐fenfluramine on food intake, pharmacological (SHU9119) or genetic (*Mc4r* null mice) blockade of one of the receptor targets for POMC peptides, the melanocortin4 receptor (MC4R), attenuated the effects of d‐fenfluramine.^42^, ^47^ The circuit was further clarified through transgenic MC4R re‐expression, which revealed that MC4Rs within the paraventricular nucleus of the hypothalamus (PVH) are sufficient for d‐fenfluramine food intake suppression.^42^ In summary, a key mechanism through which d‐fenfluramine decreases feeding is via activation of the subset of 5‐HT_2C_Rs co‐expressed with ARC POMC, which stimulates the release of POMC peptides to act at MC4Rs within the PVH.

#### 7.1.2 Sibutramine

Sibutramine (Meridia) is a 5‐HT and noradrenaline reuptake inhibitor that was prescribed for obesity treatment between 1997 and 2010 and typically produced 5%‐7% weight loss.^57^, ^58^ Patient studies indicate that the anorectic effects of sibutramine are maintained during 10 months of treatment.^59^ However, sibutramine was withdrawn from clinical use in 2010 as a result of reports associating it with increased risk of myocardial infarction and stroke in obese patients.^60^

Similar to fenfluramine, sibutramine decreases feeding and enhances satiety.^59^ In rats, it has also been reported to exert effects on energy expenditure^61^; however, in humans, the effect on energy expenditure is not apparent. Weight loss is typically associated with a lowering of energy expenditure. Although one study reported that sibutramine partially attenuated the decline in energy expenditure with weight loss in obese people (*P* = 0.09),^62^ other studies report no effect of sibutramine on energy expenditure in people.^63^, ^64^ Alongside its anorectic effects, sibutramine action also improves glycaemic control in obese people with type 2 diabetes.^58^ A randomised prospective placebo‐controlled double‐blinded study in patients with type 2 diabetes prescribed metformin indicated that patients treated with sibutramine showed significant weight loss and improved glycaemic control, and demonstrated fasting blood glucose in parallel with weight loss over the 12‐month period.^65^ Sibutramine treated patients also had reduced levels of A1c (HbA_1C_), a glycated form of haemoglobin that is used as a clinical biomarker for diabetes.^65^ Similar to d‐fenfluramine, 5‐HT_2C_Rs and POMC are implicated in the obesity therapeutic effect of sibutramine.^43^, ^66^, ^67^

Medications broadly increasing 5‐HT bioavailability are effective for treating human obesity and commonly improve insulin sensitivity and glycaemic control in obese patients with type 2 diabetes by promoting weight loss. However, the risk of off‐target effects was deemed to outweigh the therapeutic benefit of fenfluramine, d‐fenfluramine, fen‐phen and sibutramine and they were subsequently withdrawn from human treatment. Other SSRIs are still commonly prescribed for conditions such as anxiety and depression (eg, fluoxetine/Prozac). Obesity increases the risk of heart disease and stroke. This suggests that a subset of obese people with increased risk of heart disease and stroke may be more susceptible cardiovascular complications associated with 5‐HT medications.

Genetic and pharmacological research indicate that the therapeutic effect of fenfluramine, d‐fenfluramine and sibutramine is primarily achieved via 5‐HT action at brain 5‐HT_2C_Rs and this spurred drug discovery efforts toward the development of selective 5‐HT_2C_R agonists. Of the 5‐HT receptors, only 5‐HT_2C_R null mice exhibit hyperphagia and obesity and develop characteristics of type 2 diabetes.^68^, ^69^ 5‐HTR knockout studies also highlight the importance of the 5‐HT_2B_Rs in cardiac structure and function.^70^ The only 5‐HT receptor that is fatal if knocked out in mice is the 5‐HT_2B_R.^71^ Thus, a challenge during this drug discovery period was to develop a compound with high affinity for the 5‐HT_2C_Rs but with low affinity for the 5‐HT_2B_Rs.

### 7.2 5‐HT_2C_R agonists

#### 7.2.1 Preclinical 5‐HT_2C_R agonists

Preclinical *meta*‐chlorophenylpiperazine (mCPP) provided some of the first insights into the potential of a 5‐HT_2C_R agonist for obesity treatment. However, this compound was unlikely to be suitable for widespread human use because it has affinity for multiple receptors in addition to the 5‐HT_2C_R (pEC_50_ 7.00), including the 5‐HT_2B_R (pEC_50_ 7.2). Similar to former 5‐HT weight loss medications, mCPP reduces food intake and body weight in rodents ^72^ and humans.^73^ This effect appears to be primarily achieved via the 5‐HT_2C_Rs given that mCPP does not reduce feeding in mice lacking 5‐HT_2C_Rs.^68^

The first clear indication of the potential of 5‐HT medications in the direct improvement of glycaemic control was observed with mCPP. In a dietary‐induced obese mouse model of type 2 diabetes, a single treatment with mCPP rapidly improved glucose and insulin tolerance over the next 2 hours.^74^ Significant improvements in glycaemia were achieved at doses of mCPP that were too low to reduce feeding. Specifically, improvements in glycaemia were achieved at 1 mg/kg, whereas more than double of this dose (2.5 mg/kg) was required to impact feeding behaviour. A single pre‐treatment of mCPP prior to a bolus of insulin also significantly increased insulin sensitivity in muscle and liver.^74^ Similarly, prolonged treatment with mCPP improved glucose and insulin tolerance, reduced hyperinsulinaemia and decreased enzymes associated with hepatic glucose production.^74^ These beneficial effects were achieved at a dose of mCPP that was too low to impact feeding or body weight.^74^ These findings illustrated the first dissociation between the effects of a 5‐HT drug on appetite/body weight and glycaemic control/insulin sensitivity.

Research investigating the mechanism of action of mCPP and other preclinical 5‐HT_2C_R agonists (eg BVT.X and WAY161,503) on appetite and glycaemic control/insulin sensitivity also point to the melanocortin system. WAY161,503 increases the activity of ARC POMC neurones.^43^ Similarly, mCPP increases the activity of ARC POMC cells and this is achieved via a postsynaptic mechanism reliant on the presence of TRPC channels.^47^, ^75^ Illustrating the necessity of the subset of 5‐HT_2C_Rs specifically in POMC neurones, mCPP does not impact feeding in mice selectively lacking 5‐HT_2C_Rs only in POMC neurones.^76^ D'Agostino et al^44^ revealed that POMC peptides are required for the complete anorectic effect of WAY161,503 using both a transgenic knockout and a brain region selective *Pomc* CRISPR/Cas9 knockdown approach in mice. Similarly, BVT.X is ineffective at reducing food intake in MC4R knockout mice.^77^

As noted above, whole brain 5‐HT_2C_R knockout mice exhibit obesity and parameters associated with type 2 diabetes (eg, insulin resistance and impaired glucose tolerance).^60^ On a high‐fat diet, 5‐HT_2C_R knockout mice also develop hyperglycaemia.^60^ Selectively knocking out 5‐HT_2C_Rs only in POMC neurones does not promote obesity, but does cause hyperinsulinaemia, hyperglycaemia, hyperglucagonaemia and insulin resistance.^76^ These findings provide further evidence that 5‐HT_2C_Rs play a key role in glycaemic control independently of their established effect on appetite and body weight. Pharmacologically or genetically blocking one of the downstream receptor targets of POMC peptides, the MC4R, prevents the effects of mCPP on glycaemic control and insulin sensitivity.^74^

These preclinical findings highlighted the potential for a 5‐HT_2C_R agonist for the treatment of both obesity and type 2 diabetes and indicate that both effects are achieved via the melanocortin system. Follow‐up research with 5‐HT_2C_R agonist lorcaserin, as described below, reveals the different branches of melanocortin circuitry producing these effects.

#### 7.2.2 Weight loss medication lorcaserin

The USA FDA approval of the selective 5‐HT_2C_R agonist lorcaserin in 2012 represented the first weight loss medication approved by the FDA in over a decade. Lorcaserin is a full agonist at the 5‐HT_2C_R, where it exhibits 104‐fold selectivity over the 5‐HT_2B_R.^78^ Lorcaserin improves obesity by reducing food intake.^44^, ^45^, ^78^, ^79^ It does not alter energy expenditure.^80^ In addition to homeostatic feeding, lorcaserin also decreases operant responding for food reward, impulsivity and binge eating.^81^, ^82^, ^83^, ^84^ In clinical trials, lorcaserin reduced body weight by approximately 5% in obese patients.^85^, ^86^, ^87^ Lorcaserin's weight loss is characterised by a reduction of fat mass but not lean mass.^78^ In another study, lorcaserin treatment led a reduction in central adiposity compared to placebo.^88^ Trunk fat mass has been associated with a higher risk of obesity‐associated comorbidities such as cardiovascular diseases, further highlighting the potential benefits of lorcaserin treatment. These cardiometabolic health improvements associated with lorcaserin treatment have been shown to be dependent on a reduction in total atherogenic lipoproteins.^89^ A large scale, 5‐year cardiovascular analysis was performed in the CAMELLIA‐TIMI 61 trial described below.

Lorcaserin produces an improvement in glycaemic control in mouse models of type 2 diabetes independent of weight loss.^45^ Indeed, the doses of drug required to improve glycaemia (4 mg/kg) were approximately half that required to decrease food intake (7.5 mg/kg).^45^ These benefits of lorcaserin were achieved via reducing hepatic glucose production and increasing insulin sensitivity.^45^ Lorcaserin does not appear to influence insulin secretion in mice.^45^ Demonstrating the translational potential of lorcaserin for the treatment of type 2 diabetes, clinical trials in obese patients with type 2 diabetes revealed that lorcaserin improved HbA_1C_ levels and fasting glucose levels^86^ irrespective of the weight loss.^90^ Data obtained from the approximately 12,000 patient CAMELLIA‐TIMI 61 trial indicated that lorcaserin significantly reduced the risk of developing diabetes by 23% in participants without prediabetes or diabetes and by 19% in participants with prediabetes.^91^ In participants with diabetes, lorcaserin significantly lowered HbA_1C_ and the risk of diabetic microvascular complications.^91^

Analysis of the mechanism of action revealed that lorcaserin requires POMC to produce its effects on food intake.^44^ Similalrly, the glucoregulatory effects of lorcaserin are absent in brain *Pomc* null mice, but the specific re‐expression of *Pomc* only in 5‐HT_2C_Rs neurones is sufficient to restore the glycaemic actions of lorcaserin.^45^ The effects of lorcaserin on food intake, glycaemia and insulin sensitivity require downstream MC4Rs.^45^ The effects on glycaemia and insulin sensitivity, but not food intake, require the specific subset of MC4Rs expressed in preautonomic cholinergic neurones in the dorsal vagal complex and spinal cord.^45^ These data illustrate the dissociation of the circuitry through which lorcaserin modulates appetite and blood glucose/insulin sensitivity.

With regards to safety, the FDA requested a long‐term trial evaluating the potential cardiovascular effects of lorcaserin as part of a postmarketing requirement. The CAMELLIA‐TIMI 61 trial comprised a 5‐year, randomised, double‐blind, placebo controlled, multicentre trial using 473 sites in eight countries. Involving approximately 12,000 overweight or obese patients with atherosclerotic cardiovascular disease or multiple cardiovascular risk factors, CAMELLIA‐TIMI 61 comprises the largest cardiovascular outcome study for a weight loss medication to date. No significant differences between treatment groups were seen for any major adverse events.^92^, ^93^ Rather, lorcaserin promoted significant weight loss and provided further health benefits, decreasing the risk of the development of diabetes and improving diabetes and diabetic microvascular complications in participants with type 2 diabetes.^92^, ^93^ In a decision that is difficult to interpret, in 2020, the FDA concluded that the potential risks of lorcaserin outweigh its benefits. Despite the finding that lorcaserin did not increase the incidence of any serious adverse events, the FDA focussed on a small numerical difference in the incidence of malignancies during the CAMELLIA‐TIMI 61 trial: 7.7% of lorcaserin treated patients compared to 7.1% of placebo treated patients were diagnosed with some form of cancer over the 5‐year period. Given that obesity is established to increase the risk of cancer (by approximately 7.1% over 5 years as suggested by the placebo‐treated group) and that there was no statistical difference between the lorcaserin and placebo groups with respect to the incidence of malignancies, the FDA’s interpretation of the similar rates of malignancies over the 5 year monitoring period is difficult to understand. The manufacturer of lorcaserin, Eisai, voluntarily withdraw lorcaserin from the USA market for obesity treatment in February 2020. However, lorcaserin is still marketed in other countries for obesity and, in consultation with the FDA, lorcaserin is entering into a phase 3 trial (MOMENTUM 1) for Dravet syndrome, a severe form of epilepsy that first presents in infancy. Mice lacking 5‐HT_2C_Rs are prone to seizures that can be fatal,^68^ providing a rationale that a 5‐HT_2C_R agonist could be effective for treating seizures associated with Dravet syndrome.

In summary, 5‐HT_2C_R agonists are effective at improving obesity and show therapeutic benefit with respect to glycaemic control and insulin sensitivity in obese individuals with prediabetes or type 2 diabetes. Lorcaserin is also effective at reducing the onset of new type 2 diabetes. These effects on diabetes parameters are not dependent upon weight loss, indicating a parallel mechanism of action. Current frontline diabetes medications target peripheral tissues to produce a therapeutic benefit. Thus, these findings with 5‐HT_2C_R agonists represent a first indication that the brain may be harnessed to improve type 2 diabetes in humans. Lorcaserin reduces body weight by promoting satiety and also decreases motivation for food reward and impulsivity.^81^, ^82^, ^94^ It is therefore perhaps surprising that the effects on body weight are not larger. 5‐HT_2C_Rs are subjected to RNA editing events in which adenosine deaminase converts adenosine residues to inosines. These post‐transcriptional modifications can impact the efficacy of receptor to G‐protein coupling, suggesting an additional mechanism by which 5‐HT signal transduction is regulated.^95^ In a genetically engineered mouse line, increased 5‐HT_2C_R RNA editing attenuated the anorectic effect of 5‐HT_2C_R agonist WAY161,503.^96^ It is therefore possible that 5‐HT_2C_R RNA editing may impact the efficacy of 5‐HT_2C_R medications in patients. This possibility remains to be investigated.

## 8 CONCLUSIONS

Obesity and type 2 diabetes are currently a growing global health concern. The brain, acting as the principal orchestrator of feeding behaviours, hosts a complex array of networks regulating energy homeostasis. Key amongst these are 5‐HT circuits and, correspondingly, various therapeutics target components of this system, particularly the 5‐HT_2C_Rs. Weight loss commonly improves type 2 diabetes and, as such, 5‐HT obesity drugs would be expected to improve glycaemic control and insulin sensitivity in patients with type 2 diabetes. However, recent research has provided compelling evidence that 5‐HT_2C_R agonists produce effects on blood glucose and insulin sensitivity independently of weight loss. As such, this opens up a potentially new category of type 2 diabetes medications, comprising a class of drugs that target the brain.

Early 5‐HT weight loss medications (fenfluramine, d‐fenfluramine and sibutramine) alter whole body 5‐HT bioavailability and therefore influence multiple 5‐HT receptors, including those not directly involved in the regulation of ingestive behaviour and metabolism. Side effects led to their withdrawal and the emergence of the selective 5‐HT_2C_R agonist lorcaserin. Lorcaserin directly targets the principal 5‐HT receptor influencing body weight. The therapeutic efficacy of lorcaserin is associated with an improved side effect profile attributed to lack of agonism at the 5‐HT_2B_R.^85^ The clinical utility of medications such as lorcaserin has nonetheless highlighted the need to further understand the mechanisms through which their beneficial effect is achieved. Research indicates that lorcaserin requires melanocortin pathways to reduce food intake and improve glycaemic control/insulin sensitivity. Principal MC4Rs controlling feeding reside within the paraventricular nucleus of the hypothalamus,^97^, ^98^ whereas MC4Rs controlling the effects of lorcaserin on glycaemia and insulin sensitivity reside in the brainstem and spinal cord.^45^

Current weight loss medications and those in clinical trials combine different ligands to produce a greater therapeutic effect (eg, contrave: bupropion and naltrexone; quismia: phentermine and topiramate; and various emerging gut peptide combinations). Thus, another future strategy to improve the therapeutic profile of lorcaserin is combination therapy. 5‐HT_1B_R agonists CP‐94253 and RU‐24969 promote food intake reductions and this effect is blocked by pre‐treatment with the 5‐HT_1B_R antagonists.^40^, ^99^ Combined administration of sub‐anorectic doses of 5‐HT_1B_R and 5‐HT_2C_R agonists results in a synergistic reduction in food intake in mice^100^ and rats.^101^ 5‐HT_1B_Rs are primarily expressed as heteroreceptors on non‐5‐HT terminals, where they prevent the release of other neurotransmitters. Evidence suggests that a key mechanism underpinning the anorectic effects of 5‐HT_1B_Rs is via hyperpolarisation of AgRP neurones and the resultant reduction in the inhibitory tone onto ARC POMC neurones.^40^, ^100^ The combination of 5‐HT_1B_R and 5‐HT_2C_R agonists thereby increases the absolute number of POMC neurones activated compared to each agonist administered alone and this results in a correspondingly greater reduction in food intake.^102^ Whether this combination also increases the effects of lorcaserin on glycaemic control and insulin sensitivity has not yet been reported. These findings illustrate the potential of a combination of a 5‐HT_2C_R agonist with other anorectic ligands to produce an improved therapeutic profile.

Compelling genetic, pharmacological and anatomical evidence generated over the last half century points to the 5‐HT system as having a key role in the regulation of energy homeostasis and in insulin secretion from β cells. Brain 5‐HT via action at 5‐HT_2C_Rs impacts feeding, body weight, glycaemic control and insulin sensitivity. Pancreatic 5‐HT is co‐secreted from β cells where it acts at 5‐HT_1A_Rs, 5‐HT_1F_Rs and 5‐HT_2B_Rs to influence insulin secretion. The mechanisms through which 5‐HT modulates insulin sensitivity and insulin release are still not well understood and provide an exciting opportunity for future research.

## CONFLICT OF INTERESTS

The authors declare that they have no conflicts of interest.

## AUTHOR CONTRIBUTIONS

**Teodora Georgescu:** Writing – original draft; Writing – review & editing. **David Lyons:** Supervision; Writing – original draft; Writing – review & editing. **Lora K. Heisler:** Supervision; Writing – original draft; Writing – review & editing.

### PEER REVIEW

The peer review history for this article is available at https://publons.com/publon/10.1111/jne.12960.
